# Prostatic chronic inflammation and prostate cancer risk at baseline random biopsy: Analysis of predictors

**DOI:** 10.1080/2090598X.2020.1757335

**Published:** 2020-05-13

**Authors:** Alessandro Tafuri, Marco Sebben, Giovanni Novella, Marco Pirozzi, Tania Processali, Aliasger Shakir, Riccardo Rizzetto, Nelia Amigoni, Riccardo Bernasconi, Matteo Brunelli, Maria A. Cerruto, Salvatore Siracusano, Alessandro Antonelli, Walter Artibani, Antonio B. Porcaro

**Affiliations:** a Department of Urology, University of Verona, Azienda Ospedaliera Universitaria Integrata Verona, Verona, Italy; b Department of Neuroscience, Imaging and Clinical Science, Physiology and Pathophisiology, “G. D’Annunzio” University, Chieti, Italy; c USC Institute of Urology and Catherine and Joseph Aresty Department of Urology, Keck School of Medicine, University of Southern California (USC), Los Angeles, California, USA; d Department of Pathology, University of Verona, Azienda Ospedaliera Universitaria Integrata Verona, Verona, Italy

**Keywords:** Prostate cancer, prostatic chronic inflammation, prostate cancer tumour grade, prostate-specific antigen, prostate biopsy

## Abstract

**Objective:**

To evaluate predictors of prostatic chronic inflammation (PCI) and prostate cancer (PCa) in patients undergoing transperineal baseline random prostatic needle biopsies (BNB).

**Patient and methods:**

According to BNB outcomes, patients were divided into four groups: cases without PCI or PCa (Control group), cases with PCI only (PCI group), cases with PCa and PCI (PCa+PCI group) and cases with PCa only (PCa group). A multinomial logistic regression model was used to evaluate the association of clinical factors with BNB outcomes. Additionally, clinical factors associated with the risk of PCa in the overall population were investigated using a multivariable logistic regression model (univariate and multivariate analysis).

**Results:**

Overall, 945 patients were evaluated and grouped as follows: Control group, 308 patients (32.6%); PCI group, 160 (16.9%); PCa+PCI group, 45 (4.8%); and PCa group, 432 (45.7%). Amongst these, PCa was independently predicted by age (odds ratio [OR] 1.081), prostate specific-antigen level (PSA; OR 1.159), transition zone volume (TZV; OR 0.916), and abnormal digital rectal examination (DRE; OR 1.962). PCa and PCI (4.8%) were independently predicted by age (OR 1.081), PSA level (OR 1.122) and TZV (OR 0.954). In the group without PCa, the PSA level was the only factor associated with the risk of PCI when compared to the control group (OR 1.051, *P* = 0.042). Among patients with PCa, independent factors associated with the risk of only PCa compared to cases with PCA+PCI were TZV (OR 0.972) and number of positive cores (OR 1.149). In the overall population, PCI was the strongest predictor of a decreased risk of PCa (multivariate model, OR 0.212; *P* < 0.001)

**Conclusions:**

At BNB, PCI was associated with both a decreased risk of PCa and less aggressive tumour biology amongst patients with PCa. The presence of PCI on biopsy cores should be reported because of its implications in clinical practice.

**Abbreviations:**

BGG: biopsy Gleason Group; BPC: biopsy positive (cancer) cores; BMI: body mass index; FGF-2: fibroblast growth factor 2; IL: interleukin; ISUP: International Society of Urologic Pathology; NIH: National Institutes of Health; OR: odds ratio; PCa: prostate cancer; PCI: prostatic chronic inflammation; TGF: transforming growth factor; TPV: total prostate volume; TZV: transition zone volume

## Introduction

For many years, chronic inflammation has been suspected as playing a major role in the pathogenesis of cancer [1]. However, this relationship is unclear and controversial with respect to prostate cancer (PCa).

In daily clinical practice, the presence of prostatic inflammation is under-estimated [2]. Prostate biopsy may reveal different types of prostatitis, such as acute prostatic inflammation, prostatic chronic inflammation (PCI), and non-specific granulomatous prostatitis. Moreover, PCI has also been classified clinically into four categories by the National Institutes of Health (NIH) [3]. The last category, ‘asymptomatic inflammatory prostatitis’, which is also coded as ‘type IV’ is a kind of PCI detected after biopsy in patients who have no history of genitourinary tract complaints but present with increased PSA levels and/or abnormal DRE [3]. The risk of PCa has been related to multiple factors that influence the prostate microenvironment. Here, PCI may have a pivotal role in the initial phase leading to PCa. In addition, the recent development of immunotherapy and vaccines against PCa underline the pivotal role of the immune system in PCa biology [4]. Our group has previously shown an inverse association between PCI and PCa [5]. On the contrary, clinical studies have shown that a personal history of prostatitis, as well as symptom duration were significantly associated with an increased risk of PCa [6]. Further, Gurel et al. [7] reported that PCI was associated with a 30% increase in the risk of PCa.

The aim of the present study was to evaluate predictors of PCI and PCa, and the relationship between these two features in patients undergoing transperineal baseline random prostatic needle biopsies (BNB).

## Patients and methods

The study had approval from the Institutional Review Board. All patients signed an informed-consent form for data collection. From September 2010 to September 2017, the records of 1910 patients who underwent BNB for suspected prostate cancer, were retrospectively evaluated. Indications included elevated PSA levels, abnormal DRE, or abnormal imaging findings on MRI or TRUS of the prostate gland when they were performed before BNB.

Exclusion criteria were: clinically suspected acute prostatic inflammation, repeat biopsy, active surveillance, previous BPH surgery, medical treatment with 5α-reductase inhibitors, saturation biopsies and/or BNB samples with <14 cores.

### Patients’ evaluation

Age (years) and body mass index (BMI, kg/m^2^) were evaluated for each patient. PSA (ng/mL) was measured by immuno-radiometric test. Abnormal DRE findings were categorised as follows: diffuse hard prostate, discrete firm area, irregular contours, or prominent lobe asymmetry. The total prostate volume (TPV) and the transition zone volume (TZV) were measured by TRUS using the formula for an ellipsoid (length × width × height × 0.52). In all, 14 biopsy cores were taken using the standard TRUS transperineal technique. Each core was coded and related to a specific zone of the prostate. Analysis of target cores was excluded in order to avoid skewing phenomena. We did not consider PSA density (ratio between PSA and TPV) in order to avoid conflicting analysis.

### Pathological features

**Figure 1.**
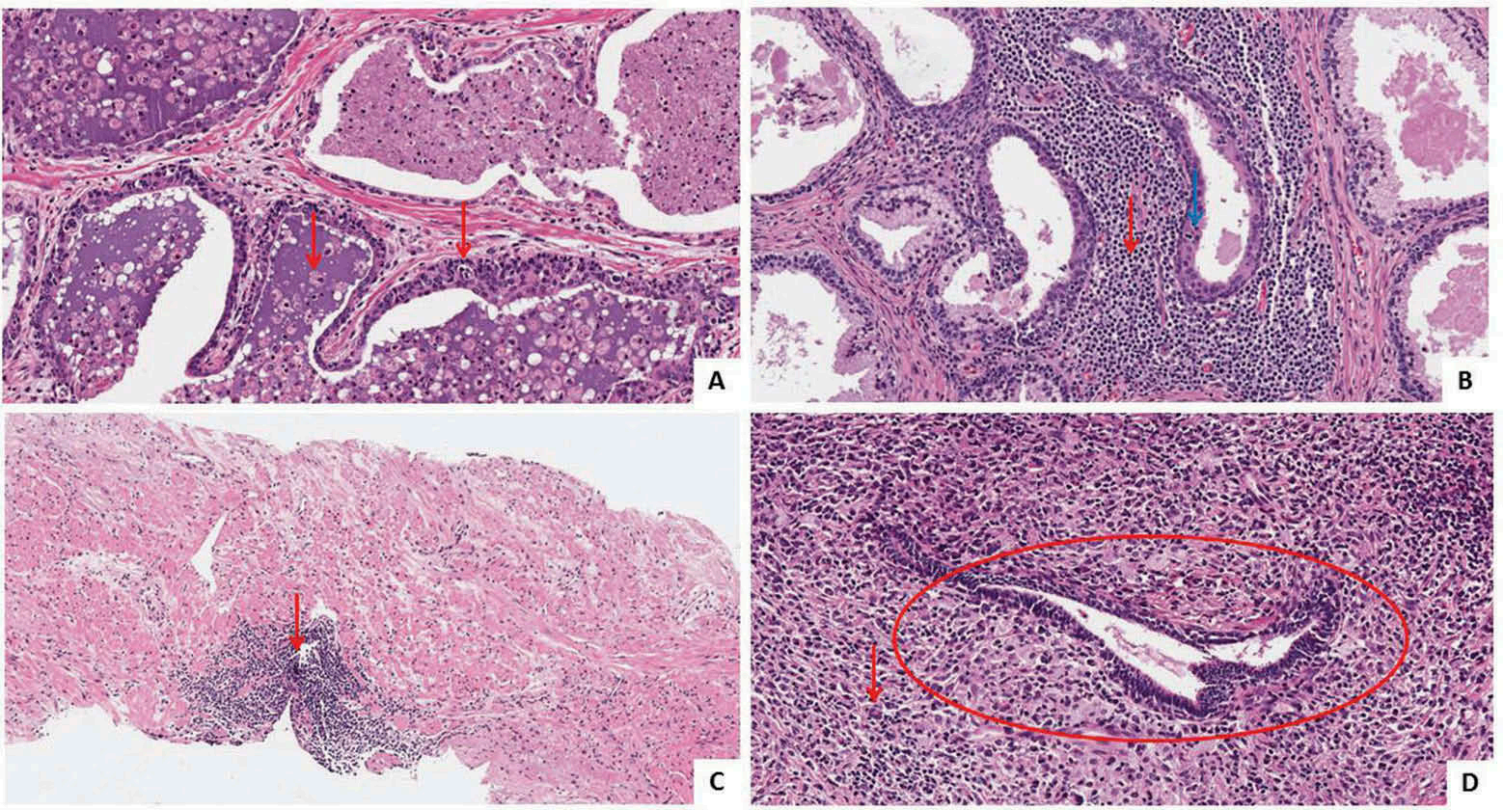
Types of prostatic inflammation diagnosed during prostate biopsy. (a) Acute prostatic inflammation: red arrows indicate neutrophil granulocytes in glandular lumen interspersed within the glandular epithelium (haematoxylin and eosin [H&E], ×20). (b) Prostatic lymphoplasmocellular chronic inflammation: the arrows indicate lymphocytes: intra-glandular (blue arrow) and extra-glandular lymphocytes (red arrow) (H&E, ×20). (c) Focal lymphoplasmocellular chronic inflammation. The red arrow indicates lymphocyte aggregation (H&E, ×10). (d) Chronic granulomatosis prostatic inflammation: The red arrow indicates a giant cell, the circle indicates a prostatic acinar gland showing a slight subversion of the form surrounded by the granulomatous-giant cell lympho-plasma reaction (H&E, ×20).

Each core was evaluated by a dedicated pathologist who systematically assessed the following features: length (mm); biopsy Gleason Group (BGG) and tumour grade group according to the International Society of Urologic Pathology (ISUP) system [8]; percentage of cancer involving each core; biopsy positive (cancer) cores (BPC); prostatic intraepithelial neoplasia; chronic inflammatory infiltrate or PCI, defined as the presence of lymphocytes, macrophages and dendritic cells or mixed cells, glandular atrophy, and atypical small acinar cell proliferation. In each core, the presence of chronic inflammation was evaluated (Figure 1). Morphologically, we focussed on type IV prostatitis, according to the NIH consensus, where the definition of prostatitis type IV [3] relates to patients who present with an elevated serum level of PSA and/or abnormal DRE and no history of genitourinary tract complaints, thus requiring a prostate biopsy to exclude PCa.

### Statistical methods

Patients were divided into four groups according to biopsy outcomes as follows: (i) negative for PCa and PCI (Control group); (ii) cores with PCI without PCa (PCI group); (iii) cores with PCa and PCI (PCI+PCA group); (iv) cores with PCa without PCI (PCa group). Summary statistics of population and subpopulations were computed.

If three or more groups were compared, differences among groups were assessed using the Kruskal–Wallis test and chi-squared test for continuous and categorical variables, respectively. Differences in continuous variables between two groups were assessed using the Mann–Whitney test. Fisher’s exact test was computed when appropriate.

The multinomial logistic regression model (univariate and multivariate analysis) assessed the association of clinical factors amongst groups.

Furthermore, clinical factors associated with the risk of PCa were investigated by multivariable logistic regression models (univariate and multivariate analysis).

The software used for the analysis was the Statistical Package for the Social Sciences (SPSS®), version 20 (SPSS Inc., IBM Corp., Armonk, NY, USA). All tests were two-sided, with a significance level of *P* < 0.05.

## Results

After applying the exclusion criteria, 945 patients were evaluated. The patients’ distribution between groups was as follows: Control group, 308 patients (32.6%); PCI group, 160 (16.9%); PCa+PCI group, 45 (4.8%); and PCa group, 432 (45.7%). The patients with PCa with or without PCI, when compared to controls and patients with PCI, were older and had higher PSA levels, lower prostate volumes (TPV and TZV), and higher rates of abnormal DRE findings. The PCa group, when compared with PCa+PCI group, had higher median values for positive cores (4 vs 2; *P* < 0.001) and higher rates of intermediate- and high-grade tumours (53.2% vs 28.9%; *P* < 0.001). Details are listed in Table 1.

**Table 1.** Associations of clinical variables in subgroups of a population including 945 patients who underwent a BNB set because of suspected PCa.

Variable	Population	Subgroups by BNB outcomes				*P*
		Control (*)	PCI	PCa+PCI	PCa	
*N* (%)	945	308 (32.6)	160 (16.9)	45 (4.8)	432 (45.7)	
Median (IQR):						
Age, years	67 (61–72)	65 (58–70)	66 (59–70.7)	69 (64–72)	69 (63–74)	<0.001
BMI, kg/m^2^	26.1 (24.2–28.1)	26 (24–27.7)	26.3 (24.2–28.7)	26.3 (23.9–29.4)	26.1 (23.9–29.4)	0.584
PSA level, ng/mL	6.2 (4.7–8.4)	5.8 (4.4–7.6)	6.6 (4.8–8.4)	6 (4.3–8.8)	6.3 (4.9–8.9)	0.003
TPV, mL	37.9 (28–51.5)	43.4 (32.3–59.8)	44 (32.3–59.5)	35 (26.8–49.3)	32 (25.5–43.8)	<0.001
TZV, mL	18 (21.1–26)	20.8 (15–30.8)	23.8 (15.8–32.8)	16.7 (12–23.8)	14.8 (10–20.8)	<0.001
DRE, *n* (%)						
normal	637 (67.4)	241 (78.2)	118 (73.8)	28 (62.2)	250 (57.9)	<0.001
abnormal	308 (32.6)	67 (21.8)	42 (26.2)	17 (37.8)	182 (42.1)	
BPC, *n*, median (IQR)				2 (2–5.5)	4 (2–7)	<0.001(*)
BGG, *n* (%)						
BGG = 1				32 (71.1)	202 (46.8)	<0.001
BGG >1				13 (28.9)	230 (53.2)	

BGG: biopsy tumour grade group; BMI: body mass index; BPC: biopsy positive cores; PCa: prostate cancer; PCI: prostate chronic inflammation; (*): no PCA and no PCI; TPV: total prostate volume; TZV: transition zone volume; Mann–Whitney *U*-test.

Among groups without PCa, the PSA level was the only predictor of PCI compared to the controls (odds ratio [OR] 1.051, 95% CI 1.002–1.082; *P* = 0.042) (Supplementary Table S1).

Independent clinical factors associated with the risk of PCa+PCI compared to controls were age (adjusted OR 1.065, 95% CI 1.023–1.109; *P* = 0.002), PSA level (adjusted OR 1.122, 95% CI 1.040–1.211; *P* = 0.003), and TZV (inverse association; adjusted OR 0.954, 95% CI 0.927–0.982; *P* = 0.002).

Among patients with PCI on biopsy, the TZV was the only factor associated with the simultaneous presence of PCa and PCI (inverse association; OR 0.965, 95% CI 0.939–0.993; *P* = 0.013) (Supplementary Table S2).

Independent clinical factors associated with PCa compared with controls (Table 2) were age (OR 1.081), PSA level (OR 1.159), TZV (OR 0.916), and abnormal DRE (OR 1.196) (all *P* < 0.001).

**Table 2.** Variables associated with PCa (*n* = 432) compared to controls (*n* = 308).

Variable	Univariate model		Multivariate model		Multivariate model (adjusted OR)	
	OR (95% CI)	*P*	OR (95% CI)	*P*	OR (95% CI)	*P*
Age	1069 (1.049–1.090)	<0.001	1080 (1.057–1.104)	<0.001	1081 (1.058–1.104)	<0.001
BMI	1022 (0.976–1.069)	0.354				
PSA	1087 (1.047–1.130)	<0.001	1163 (1.106–1.223)	<0.001	1159 (1.103–1.219)	<0.001
TPV	0.960 (0.951–0.969)	<0.001	0.980 (0.958–1.003)	0.092		
TZV	0.937 (0.923–0.951)	<0.001	0.942 (0.908–0.976)	0.001	0.916 (0.901–0.932)	<0.001
DRE						
Normal	Ref.					
Abnormal	2619 (1.880–3.647)	<0.001	1973 (1.340–2.799)	<0.001	1962 (1.359–2.832)	<0.001

BMI: body mass index; CI: confidence interval of OR; OR: odds ratio; PCa: prostate cancer; PCI: prostatic chronic inflammation; TPV: total prostate volume; TZV: transition zone volume.

Independent factors predicting patients with PCa compared to patients with PCI (Table 3) were age (OR 1.072) and TZV (OR 0.913) (both *P* < 0.001).

**Table 3.** Variables associated with PCa (*n* = 432) compared to PCI (*n* = 160).

Variable	Univariate model		Multivariate model		Multivariate model (adjusted OR)	
	OR (95% CI)	*P*	OR (95% CI)	*P*	OR (95% CI)	*P*
Age	1.052 (1.029–1.076)	<0.001	1.069 (1.042–1.096)	<0.001	1.072 (1.046–1.099)	<0.001
BMI	1.002 (0.948–1.059)	0.944				
PSA level	1.034 (0.993–1.077)	0.104				
TPV	0.959 (0.949–0.969)	<0.001	1.002 (0.977–1.027)	0.602		
TZV	0.930 (0.915–0.945)	<0.001	0.913 (0.879–0.949)	<0.001	0.634 (0.986–0.930)	<0.001
DRE						
Normal	Ref.					
Abnormal	2.045 (1.370–3.053)	<0.001	1.278 (0.959–2.277)	0.077		

CI: confidence interval of OR; OR: odds ratio; PCa: prostate cancer; PCI: prostatic chronic inflammation; TPV: total prostate volume; TZV: transition zone volume.

On univariate analysis, among all patients with PCa (*n* = 432), TPV (OR 0.975, *P* = 0.005), TZV (OR 0.963, *P* = 0.011), BPC (OR 1.164, *P* = 0.011), BGG 2/3 (OR 2.463, *P* = 0.013) and BGG >3 (OR 4.673, *P* = 0.038) were associated with a lower probability of having PCI with PCa. BPC and BGG were not entered simultaneously in the multivariate analysis because they were significantly correlated, so we evaluated multivariate model I and II (model I considered TZV and BPC; model II considered TZV and BGG). In multivariate model I, a smaller TZV (OR 0.972, *P* = 0.047) and higher BPC (OR 1.149, *P* = 0.021) were associated with a higher probability of having only PCa in the biopsy specimen. In multivariate model II, TZV (OR 0.967, *P* = 0.009), BGG 2/3 (OR 2.311, *P* = 0.023) and BGG >3 (OR 4.651, *P* = 0.039) predicted the presence of PCa without PCI (Table 4).

**Table 4.** Variables associated with PCa (*n* = 432) compared to PCa+PCI (*n* = 45).

Variable	Univariate model		Multivariate model I		Multivariate model II	
	OR (95% CI)	*P*	OR (95% CI)	*P*	OR (95% CI)	*P*
Age	1.015 (0.976–1.054)	0.460				
BMI	0.982 (0.895–1.077)	0.982				
PSA level	1.011 (0.949–1.078)	0.726				
TPV	0.975 (0.958–0.992)	0.005				
TZV	0.963 (0.936–0.991)	0.011	0.972 (0.946–1.000)	0.047	0.672 (0.959–0.994)	0.009
DRE						
Normal	Ref.					
Abnormal	1.199 (0.637–2.256)	0.399				
BPC	1.164 (1.035–1.308)	0.011	1.149 (1.022–1.293)	0.021		
BGG						
BGG = 1	Ref.					
BGG 2/3	2.463 (1.205–5.032)	0.013			2.311 (1.124–4.751)	0.023
BGG >3	4.673 (1.088–20.077)	0.038			4.651 (1.078–20.063)	0.039

BGG: biopsy tumour grade group; BPC: biopsy positive cores; CI: confidence interval of OR; OR: odds ratio; PCa: prostate cancer; PCI: prostatic chronic inflammation; TPV: total prostate volume; TZV: transition zone volume.

Additionally, clinical factors associated with the risk of PCa in the general population (945 patients who underwent a first standard BNB set) were investigated using the multivariate logistic regression model (Supplementary Table S3). Among those, PCI on multivariate analysis remained the strongest predictor of a decreased risk of PCa (OR 0.212, *P* < 0.001).

## Discussion

### PCI and PCa

We evaluated the predictors of PCI and PCa in a large cohort of patients undergoing BNB. PCI decreased the risk of PCa; moreover, among patients with PCa (with or without PCI), a small TZV, high BPC and the presence of ISUP Grade >1 were associated with a lower probability of having PCI.

In the last few years there has been great interest in the factors that influence PCa biology. Among these, the detection of PCI at BNB has become a pivotal feature, which still remains controversial and unresolved [9].

We evaluated the main features of clinical studies examining the relationship of PCI and PCa detection at BNB and compared them with the results of the present study (Supplementary Table S4). The study designs were prospective or retrospective. The association between PCI and PCa risk was inverse in four studies and none in three [10–16]. With BNB, the detection of only PCI is frequent and variable, ranging from 6.7% to 69%. Moreover, the detection of PCI with PCa may range from 0.3% to 37.4%. In our present study, the detection rate of PCI+PCa was 4.8%, which is close to the rates reported by Hu et al. [16] (6.2%) and Amini et al. [10] (5.7%). In all studies, the main indications for an initial BNB were increased serum PSA levels, abnormal DRE, or both. Hu et al. [16] first reported that PCI is significantly associated with PCa risk at BNB. Karakiewicz et al. [13] found an independent association of clinical factors (positive for age, PSA level, DRE; negative for prostate volume) and PCa, but they did not evaluate these associations with PCI or for PCa with PCI. Although Bassett et al. [11] reported that only prostate volume was an independent predictor of PCI. Amini et al. [10] found that age, PSA level, TZV and PCI were independently associated with PCa. In addition, Moreira et al. [17] reported a negative association between PCI and PCa in patients undergoing re-biopsy when PCI was detected at BNB. They concluded that the presence of PCI at BNB was a negative predictor of PCa risk at re-biopsy. Furthermore, a recent meta-analysis has shown that the presence of PCI in biopsy cores is associated with a decreased risk of PCa [9]. Our previous investigations showed an inverse association between PCI and PCa. Also, we found that the risk of high-grade tumours is decreased in patients with PCa in whom PCI is present when compared to those with PCa but without PCI [5,18,19]. On the other hand, Gurel et al. [7] have shown a positive association between PCI and risk of PCa, as well as with high-grade disease.

In the present study, we found that high BPC was associated with a lower probability of having PCI but a higher probability of having a more aggressive PCa. These results are concordant with our previous experience, demonstrating that more positive cores were associated with more aggressive disease [20,21].

Further, in the present study we found that the TZV, that strongly influences the TPV, is inversely correlated to PCa risk. Indeed, it is known that prostate volume has an inverse correlation with PCa risk [22,23]. However, there may be a greater chance of accurately sampling a cancer lesion with the biopsy needle in patients with smaller prostates compared to patients with larger prostates with similar lesions. This phenomenon could also explain the lower cancer detection rates in large prostates. This hypothesis is debated and has not been confirmed in the literature [24].

### Biological hypothesis

Prostate cancer, as well as BPH, is considered a chronic condition derived from different local and systemic situations. It is known that many factors can induce chronic inflammation of the prostate gland and PCI can have an important role in PCa and BPH physiopathology. In addition, different systemic factors such as androgen and oestrogen hormonal levels, can influence cellular differentiation and cytokine secretion in prostatic inflammatory tissue and different cytokines can have multiple effects on prostatic cells [25]. In the last few years, many studies have reported an association between serum androgen levels and PCa biology [26]. In theory, changes in serum androgen levels in middle-aged men can be a catalyst for changes in the prostatic microenvironment and PCa induction [27]. These changes and associated systemic conditions and external factors, could influence the prostatic inflammatory tissue to modify the cellular infiltrate and local cytokine secretion. In particular, systemic changes could stimulate the production of interleukin (IL)-2, −4, −13, −17, −23, transforming growth factor β (TGF-β) and fibroblast growth factor 2 (FGF-2) associated with benign conditions. The production of other kinds of cytokines as IL-6, −8 or TGF-α, on the contrary, can contribute to PCa induction and growth [25].

In our present study, PCI was associated with both a decreased risk of PCa and less aggressive tumour biology amongst patients with PCa. The results of the present study, as well as the evidence provided by other studies (Supplementary Table 4), can be explained by the secretion of PCa protective cytokines. On the other hand, when PCI was associated with PCa or more aggressive cancer biology, alternate pathways, with different cytokines are implicated. Supporting our hypothesis, there has been recent interest in prostate anti-cancer vaccines and immunotherapies focussed on empowering the immune system to overcome PCa. Vaccines and/or immunotherapies aim to stimulate the immune system to activate an appropriate immune-mediated response against malignant cells [4,28].

### Limitations, strengths and application to the daily clinical practice

Our present study has several limitations. First, it is a retrospective study with all biases of these kinds of investigations. Second, the TPV and TZV could not be assessed by pathology; further, volume evaluations were performed using the ellipsoid-TRUS method that has been shown to have a non-negligible intra- and inter-observer variability [29]. Third, patients with a PCI microenvironment are more likely to undergo BNB because of the condition is associated with increased PSA levels, which may be a source of selection bias. However, we have shown that the PSA level was not identified as an independent factor by our multivariate models in predicting patients with PCa.

In the present patient cohort, we did not adopt any evaluation to exclude PCI before biopsy. However, in a recent experience, we demonstrated that prostate volume index, defined as the ratio of the TZV to the peripheral zone volume, was able to differentiate between PCI and PCa in patients with a normal DRE and PSA level <10 ng/mL, who underwent BNB for suspected PCa, but additional higher level studies are needed to confirm these findings before introducing this parameter into daily clinical practice [30].

Additionally, in the Control group patients, the presence of PCa was not immediately further assessed but they were maintained on a clinical follow-up regimen. Specifically, patients who had a negative biopsy with a persistent suspicion of PCa underwent re-biopsy with prior multiparametric MRI (if not previously performed) within 4 months of the BNB.

Although, our present study has the above limitations, it also has strengths that should be outlined. First, it is a large retrospective study confirming the results of other large trials. Second, it is the first study that shows the association of clinical factors with BNB outcomes with stratification of patients into four defined groups. Third, it gives evidence of the importance of measuring the TZV, which is closely related to the spectrum of prostate diseases.

Furthermore, our present study can have important implications in clinical practice. First, pathologists should report the presence of PCI at BNB because this can be a critical issue for the future management of patients on active surveillance. In fact, the presence of PCI+PCA in the BNB can change the time between second biopsy and identify patients with a lower risk of upgrading to more aggressive PCa. Second, we proposed a hypothesis that could be the basis for future investigations that could change clinical practice. Clinical and preclinical studies are mandatory to confirm our present results and hypothesis.

## Conclusions

At BNB, PCI is associated with both a decreased risk of PCa and less aggressive tumour biology amongst patients with PCa. The presence of PCI on biopsy cores should be reported because of its implications in clinical practice.

## Supplementary Material

Supplemental Material
