# Supplementary material for: Prostatic chronic inflammation and prostate cancer risk at baseline random biopsy: Analysis of predictors
Source: Arab J Urol. 2020 May 13;18(3):148–54. doi: 10.1080/2090598X.2020.1757335 (PMC7473292; doi:10.1080/2090598X.2020.1757335)
Supplement: Supplemental Material [file TAJU_A_1757335_SM8821.zip › TAJU-2019-0164SupplementaryTable S3ed.docx]

**Supplementary Table S3 Univariate and multivariate analysis of clinical factors associated with prostate cancer risk.**

|  |  |  | **Univariate model** | | **Multivariate model** | |
| --- | --- | --- | --- | --- | --- | --- |
| **Clinical factors** | **No PCa** | **PCa** | **OR (95% CI)** | ***P*** | **OR (95% CI)** | ***P*** |
| Median (IQR) |  |  |  |  |  |  |
| Age, years | 65 (59–70) | 69 (63–74) | 1.062 (1.044–1.079) | <0.001 | 1.074 (1.053–1.096) | <0.001 |
| BMI, kg/m^2^ | 26.1(24–28.1) | 26.1 (24.2–28.4) | 1.017 (0.977–1.058) | 0.411 |  |  |
| PSA, level, ng/mL | 6.0 (4.5–8) | 6.3 (4.9–8.9) | 1.065 (1.033–1.098) | <0.001 | 1.138 (1.089–1.190) | <0.001 |
| TPV, mL | 43.5 (32.3–59.8) | 32.8 (26–43.9) | 0.963 (0.955–0.971) | <0.001 | 0.988 (0.968–1.009) | 0.255 |
| TZV, mL | 21.7 (15–31.6) | 14.9 (10.1–21) | 0.939 (0.927–0.952) | <0.001 | 0.940 (0.910–0.971) | <0.0001 |
| *N* (%) |  |  |  |  |  |  |
| DRE |  |  |  |  |  |  |
| Normal | 359 (76.7) | 278 (58.3) | 1 Ref. |  | 1 Ref. |  |
| Abnormal | 109 (23.3) | 199 (41.7) | 2.358 (1.780–3.123) | <0.001 | 1.817 (1.303–2.534) | <0.001 |
| PCI |  |  |  |  |  |  |
| Absent | 308 (65.8) | 432 (90.6) | 1 Ref. |  | 1 Ref. |  |
| Present | 160 (34.2) | 45 (9.4) | 0.201 (0.140–0.288) | <0.001 | 0.212 (0.141–0.318) | <0.001 |

BMI: body mass index; CI: confidence interval of OR; OR: odds ratio; PCa: prostate cancer; PCI: prostatic chronic inflammation; TPV: total prostate volume; TZV: transition zone volume.
