# Supplementary material for: Prostatic chronic inflammation and prostate cancer risk at baseline random biopsy: Analysis of predictors
Source: Arab J Urol. 2020 May 13;18(3):148–54. doi: 10.1080/2090598X.2020.1757335 (PMC7473292; doi:10.1080/2090598X.2020.1757335)
Supplement: Supplemental Material [file TAJU_A_1757335_SM8821.zip › TAJU-2019-0164SupplementaryTableS1ed.docx]

**Supplementary Table S1 Factors associated with PCI (*n* = 160) compared to controls (*n* = 308).**

| **Factors** | **Univariate model** | ***P*** |
| --- | --- | --- |
|  | **OR (95% CI)** |  |
| Age | 1.016 (0.993–1.040) | 0.181 |
| BMI | 1.020 (0.961–1.082) | 0.518 |
| PSA | 1.051 (1.002–1.082) | 0.042 |
| TPV | 1.002 (0.994–1.010) | 0.662 |
| TZV | 1.008 (0.996–1.020) | 0.184 |
| DRE |  |  |
| Normal | Ref. |  |
| Abnormal | 1.280 (0.821–1.996) | 0.276 |

BMI: body mass index; CI: confidence interval of OR; OR: odds ratio; PCI: prostatic chronic inflammation; TPV: total prostate volume; TZV: transition zone volume.
