# Supplementary material for: Prostatic chronic inflammation and prostate cancer risk at baseline random biopsy: Analysis of predictors
Source: Arab J Urol. 2020 May 13;18(3):148–54. doi: 10.1080/2090598X.2020.1757335 (PMC7473292; doi:10.1080/2090598X.2020.1757335)
Supplement: Supplemental Material [file TAJU_A_1757335_SM8821.zip › TAJU-2019-0164SupplementaryTableS2ed.docx]

**Supplementary Table S2 Factors associated with simultaneous presence of PCa and PCI (*n* =45) compared to PCI alone (*n* = 160).**

| **Factors** | **Univariate model** | ***P*** |
| --- | --- | --- |
|  | OR (95% CI) |  |
| Age | 1.037 (0.995–1.081) | 0.082 |
| BMI | 1.021 (0.924–1.128) | 0.687 |
| PSA | 1.023 (0.953–1.097) | 0.537 |
| TPV | 0.983 (0.966–1.001) | 0.064 |
| TZV | 0.965 (0.939–0.993) | 0.013 |
| DRE |  |  |
| Normal | Ref. |  |
| Abnormal | 1.706 (0.849–3.849) | 0.134 |

BMI: body mass index; CI: confidence interval of OR; OR: odds ratio; PCa: prostate cancer; PCI: prostatic chronic inflammation; TPV: total prostate volume; TZV: transition zone volume.
