# Supplementary material for: Prostatic chronic inflammation and prostate cancer risk at baseline random biopsy: Analysis of predictors
Source: Arab J Urol. 2020 May 13;18(3):148–54. doi: 10.1080/2090598X.2020.1757335 (PMC7473292; doi:10.1080/2090598X.2020.1757335)
Supplement: Supplemental Material [file TAJU_A_1757335_SM8821.zip › TAJU-2019-0164SupplementaryTableS4ed.docx]

**Supplementary Table S4 Clinical studies investigating the association between PCI and PCa risk at first biopsy.**

| **Reference** | **Year** | **Study** | | | | | **Subgroups by histology outcomes, *n* (%)** | | | **Association** |
| --- | --- | --- | --- | --- | --- | --- | --- | --- | --- | --- |
|  |  | **Period** | **Design** | **Cases, *n*** | **Cores, *n*** | **Control, *n* (%)*** | **PCI** | **PCa+PCI** | **PCa** | **PCI/PCa** |
| Hu et al. [16] | 1998 | NR | Prospective | 388 | 6 | 168 (43.3) | 91 (23.5) | 24 (6.2) | 105 (27.1) | Inverse |
| MacLennan et al. [15] | 2006 | NR | Prospective | 177 | NR | 23 (13.0) | 115 (65.0) | 29 (16.4) | 10 (5.6) | None |
| Benedetti et al. [14] | 2016 | 2010–2013 | Prospective | 203 | 8–12 | 32 (15.8) | 81 (39.9) | 76 (37.4) | 14 (6.9) | None |
| Karakiewicz et al. [13] | 2007 | 1990–1998 | Prospective | 4526 | 6 | 2592 (57.3) | 301 (6.7) | 46 (1.0) | 1587 (35.1) | Inverse |
| Abdel-Meguid et al. [12] | 2009 | 2003–2008 | Retrospective | 214 | 6 | 84 (39.3) | 54 (25.2) | 33 (15.4) | 43 (20.1) | None |
| Bassett et al. [11] | 2009 | 1990–2004 | Retrospective | 655 | 6–12 | 404 (61.7) | 92 (14.0) | 2 (0.3) | 157 (24.0) | Inverse |
| Amini et al. [10] | 2015 | 2005–2015 | Retrospective | 2207 | 12 | 884 (40.1) | 794 (36) | 126 (5.7) | 403 (18.3) | Inverse |
| Present study | 2019 | 2010–2017 | Retrospective | 945 | 14 | 308 (32.6) | 160 (16.9) | 45(4.8) | 432 (45.7) | Inverse |

*: histology negative for PCI and PCa; PCI/PCa, association between PCI and PCa risk; NR, not reported.
